# Preharvest Methyl Jasmonate Treatment Increased Glucosinolate Biosynthesis, Sulforaphane Accumulation, and Antioxidant Activity of Broccoli

**DOI:** 10.3390/antiox11071298

**Published:** 2022-06-29

**Authors:** Junwei Wang, Shuxiang Mao, Mantian Liang, Wenxia Zhang, Fangzhen Chen, Ke Huang, Qiuyun Wu

**Affiliations:** 1 College of Horticulture, Hunan Agricultural University, Changsha 410128, China; junweiwang87@126.com (J.W.); 18374878831@163.com (S.M.); liangmt0115@163.com (M.L.); z2574272943@163.com (W.Z.); cc9980705@163.com (F.C.); 2 Engineering Research Center for Horticultural Crop Germplasm Creation and New Variety Breeding, Ministry of Education, Changsha 410128, China; 3 Key Laboratory for Vegetable Biology of Hunan Province, Changsha 410128, China

**Keywords:** broccoli, MeJA, glucosinolate, sulforaphane, antioxidant activity

## Abstract

Broccoli is becoming increasingly popular among consumers owing to its nutritional value and rich bioactive compounds, such glucosinolates (GSLs) and hydrolysis products, which are secondary metabolites for plant defense, cancer prevention, and higher antioxidant activity for humans. In this study, 40 μmol/L methyl jasmonate (MeJA) was sprayed onto broccoli from budding until harvest. The harvested broccoli florets, stem, and leaves were used to measure the contents of GSLs, sulforaphane, total phenolics, and flavonoids, as well as myrosinase activity, antioxidant activity, and gene expression involved in GSL biosynthesis. The overall results revealed that GSL biosynthesis and sulforaphane accumulation were most likely induced by exogenous MeJA treatment by upregulating the expression of *CYP83A1*, *SUR1*, *UGT74B1*, and *SOT18* genes. Exogenous MeJA treatment more remarkably contributed to the increased GSL biosynthesis in broccoli cultivars with low-level GSL content (Yanxiu) than that with high-level GSLs (Xianglv No.3). Moreover, MeJA treatment had a more remarkable increasing effect in broccoli florets than stem and leaves. Interestingly, total flavonoid content substantially increased in broccoli florets after MeJA treatment, but total phenolics did not. Similarly, 2,2-diphenyl-1-picrylhydrazyl (DPPH) radical scavenging capacity, trolox-equivalent antioxidant capacity (ABTS), and ferric-reducing antioxidant power (FRAP) were higher in broccoli floret after MeJA treatment. In conclusion, MeJA mediated bioactive compound metabolism, had positive effects on GSL biosynthesis, sulforaphane, and flavonoids accumulation, and showed positive correlation on inducing higher antioxidant activities in broccoli floret. Hence, preharvest supplementation with 40 μM MeJA could be a good way to improve the nutritional value of broccoli florets.

## 1. Introduction

Plant-based foods provide people with basic nutrition and have many health care values owing to their rich bioactive compounds [1]. With the promotion of “Healthy China Action,” people’s consumption habits have gradually changed from nutrition-oriented to health-oriented, and horticultural products rich in bioactive substances have attracted increasing attention from consumers. Cruciferous vegetables are often of interest as nutritious foods since they are a source of bioactive compounds, including glucosinolates, phenolics, flavonoids, and vitamin C [2,3]. Recent reports showed that the high radical scavenging activity of cruciferous vegetables benefits from these bioactive compounds [4,5].

Broccoli (*Brassica oleracea var. italica*) is a commonly consumed vegetable belonging to cruciferous vegetables, which are rich in dietary fiber, vitamins, minerals, and bioactive compounds [6]. Similar to other cruciferous vegetables, the unique flavor of broccoli is due to the presence of glucosinolates (GSLs) and degradation products. GSLs are sulfur-rich secondary metabolites derived from glucose and amino acids. The hydrolysis products of GSLs play important roles in plant defense and exhibit biological activities that can benefit human health [7,8]. Brassicaceae vegetables, especially broccoli, are rich sources of GSLs [9]. Recent studies have shown that the consumption of broccoli and its phytochemicals are associated with reduced risks of metabolic syndrome and some types of cancer [10], especially the glucoraphanin hydrolysis product--sulforaphane which would induce antioxidant levels in the human body [11]. Based on the structure of the precursor amino acid, GSLs are usually divided into three groups: aliphatic, indolic, and aromatic GSL, which are derived from methionine, tryptophan, and phenylalanine or tyrosine, respectively [12]. GSL biosynthesis in plants occurs through three independent stages: (i) chain elongation of precursor amino acids, (ii) core structure synthesis, and (iii) secondary modifications and functionalizations of the side chain [13]. Several gene families (e.g., *BCAT*s, *MAM*s, *CYP*s, and *SOT*s) participate in GSL biosynthesis [12]. The branched-chain amino acid aminotransferase (BCATs) and methylthioalkylmalate synthase (MAMs) are the core enzyme for stage (i), which raises a 2-oxo acid to precursor amino acid and makes 2-oxo acid condensation with acetyl-CoA, respectively, with a series of subsequent reactions, the product is a 2-oxo acid which elongated by a single methylene group (–CH_2_–) [12]. Further, the cytochromes P450(P450s) and sulfotransferases (SOTs) are the crucial enzymes for stage (ii), the CYP79 family converted precursor amino acids to aldoximes, next, CYP83 family oxidized aldoximes to activated compounds (either nitrile oxides or aci-nitro compounds). After, thiohydroximates are in turn S-glucosylated to form desulfoglucosinolates, which are sulfated by the sulfotransferases SOT17 and 18 to form glucosinolates. Finally, the stage (iii) of secondary modifications lead to a large extent biological activity of glucosinolates. Moreover, R2R3-MYB transcription factors (e.g., *MYB28* and *MYB34*) play important regulatory roles in the biosynthesis pathway [14].

Polyphenols and flavonoids as important natural antioxidants act as free radical scavengers against harmful oxidants, such as reactive oxygen (ROS) and reactive nitrogen species (RNS) [15]. The balance of ROS and RNS is maintained by oxidants, and their imbalance would cause some chronic diseases, such as diabetes, cancers, and cardiovascular ailments [16]. Antioxidants also induced antioxidant enzymes and activate signaling pathway of Nrf2/ARE (nuclear transcription factor-erythroid 2 related factor/antioxidant response element) for reducing cellular oxidative damage [17]. Broccoli growth and development are affected by many factors, such as genotype, edible parts, exogenous hormone, environment factors, and insect feeding, which affect bioactive compound metabolism. Simple and effective agricultural practices have been used to promote GSL, polyphenol, or flavonoid biosynthesis for high-nutrition broccoli with abundant bioactive compounds [18,19,20].

Methyl jasmonate (MeJA), like jasmonic acid (JA), is a signal molecule released when plants are attacked by insects or from physical wounds [21]. When plants are attacked by herbivores, the JA pathway triggers the herbivorous defense system and activates a series of physiological events, one of which is GSL biosynthesis. Previous studies found that exogenous MeJA treatment leads to higher GSL biosynthesis in Brassicaceae vegetables [22], and enhance the indole GSLs (IGS) contents of broccoli florets [20,23]. However, little information or contrary results have been reported in terms of the effect of MeJA aliphatic GSLs (AGS) or sulforaphane. For example, the AGS content of broccoli floret remarkably increases after MeJA treatment in one study [19], but remarkably decreases in another study [24]. Preharvest MeJA treatment also induces polyphenol and flavonoid accumulation [1,25], which are beneficial to improve their antioxidant properties and nutritional value. Hence, MeJA is often applied before harvest as a certified safe compound for all foods.

The effects of exogenous MeJA on bioactive compound biosynthesis (including GSL, polyphenols and flavonoids) in various kinds of Brassicaceae vegetables are widely reported [26,27,28], but information on AGS involvement and even sulforaphane biosynthesis is limited. In the present study, the broccoli cultivars ‘Yanxiu’ and ‘Xianglv No. 3’ were treated with 40 μmol/L MeJA to study the variation characteristics of GSL and sulforaphane contents and the antioxidant activities in florets, stem, and leaves. The research results were used to explore the way for the high-quality cultivation of broccoli.

## 2. Materials and Methods

### 2.1. Plant Material

Experiments were conducted in a greenhouse at Hunan Agriculture University (latitude, 27.55° N; longitude, 113.92° E), Changsha, China, from 25 August 2019 to 16 January 2020. *Brassica oleracea* ‘Yanxiu’ and ‘Xianglv No. 3’ seeds were sown in plastic trays (50 cells, one seedling per cell) containing substrates (peat, perlite, vermiculite, 2:1:1) and grown in a natural-climate greenhouse. After the fourth leaf was fully developed, the plants were randomly transferred into a plastic pot containing 10 L of substrates (peat, perlite, vermiculite, 2:1:1) on 2 October 2019. A total of 300 plants were used in this experiment. After 1 week of preculturing, the seedlings were fertilized with one-strong of Hoagland’s nutrient solution with 7-day intervals until harvesting. After the budding stage, two experimental treatments were conducted as follows: (1) control, in which plants were sprayed with distilled water; and (2) MeJA (Sigma Aldrich, MO, USA), in which plants were sprayed with 40 μmol/L MeJA according to previous research results [29], which was sprayed onto each plant at 9:00 am for 4-day intervals, and applied for 10 times from budding stage to floret harvest stage, the last treatment at floret harvest stage and the floret diameter is 10–15 cm. The plants were organized in a complete randomized-block design with three replicates per treatment. The sample time were separate as 0, 1 h, 3 h, 6 h, 12 h, and 24 h, as we found that the samples of after treatment 12 h possessed highest GRA concentration level, so the samples which treated after 12 h were analyzed the whole parameter. Broccoli floret, stem, and leaves were sampled at 12 h after the last treatment. The samples were immediately frozen in liquid nitrogen and stored at −80 °C to analyze gene expression, myrosinase activity, and total antioxidant capacity. The others were dried in a freeze dryer to analyze GSL, sulforaphane, total phenolics, flavonoid content, and antioxidant activities.

### 2.2. Glucosinolate Extraction and Quantification

Total GSL extraction and quantification were performed by a previously described method with minor modifications [30]. Boiling 70% methanol was initially used to soak the freeze-dried sample powder for 20 min in a 75 °C water bath, and then 100 μL of sinigrin (5 mmol/L, internal standard) was added. After cooling, the sample was added with barium acetate and centrifuged at 12,000 rpm at 4 °C for 10 min. The supernatant was collected and re-extracted residues with 70% methanol twice. Desulfo-GSLs were adsorbed by anion-exchange chromatography through a DEAE-Sephadex A-25 column (diameter, 10 mm; maximum velocity, 475 cm/h; particle size, 40–200 μm; filtration molecular weight, 20,000). High-performance liquid chromatography (HPLC; Agilent, CA, USA) was used to analyze desulfo-GSLs. The following linear gradient program of chromatography at 30 °C was performed: 0% to 20% methanol for 20 min, 20% to 30% over 5 min, isocratic elution for 40% for 10 min, and 90% for 3 min. Moreover, the flow rate was 1 mL/min. The diode-array detector at 229 nm was used to detect desulfated GSLs. Then, GSL content was determined using desulfated GSLs and measured (expressed in μmol/g).

### 2.3. Measurement of Glucoraphanin Content, Sulforaphane Content, and Myrosinase Activity

Sulforaphane content was determined as previously described with minor modifications [31]. In a typical procedure, the powder of fresh broccoli samples was ground in liquid nitrogen and hydrolyzed with 4 mL of deionized water at room temperature for 4 h. Dichloromethane was used to extract sulforaphane at room temperature, after which the mixture was filtered with anhydrous sodium sulfate. Then, the filtered solution was dried in a vacuum using a rotary evaporator at 38 °C. Acetonitrile dissolved the products, and a 0.45 μm membrane filter was used to filter the solution into an autosampler vial. Agilent 1260 Series HPLC was used to determine sulforaphane content with C18 reversed-phase column. The chromatography program was as follows: isocratic elution program, 80% water and 20% acetonitrile; column temperature, 30 °C; wavelength, 209 nm; flow rate, 1 mL/min; time, 35 min; and injection volume, 10 μL. Sulforaphane content was calculated with a standard curve.

Myrosinase activity-detection protocol as previously described by Guo et al. [32]. About 100 mg of fresh broccoli sample was homogenized in 1 mL of 10 mM potassium phosphate buffer (pH, 7.2; 1 mM EDTA; containing 3 mM DTT, and 5% glycerol). Next, the mixture was centrifuged at 4 °C for 12,600 rpm for 20 min, and the supernatant was collected. The content of glucose released from sinigrin, which was hydrolyzed by enzyme, represented myrosinase activity. A glucose assay kit (Shanghai Zhuocai Biotech Inc., Shanghai, China) was used to determine glucose content using an ultraviolet spectrophotometer. About 100 μL of enzyme solution was mixed in 200 μL of 2 mM sinigrin (Sigma Aldrich, MO, USA), and a mixture with no sinigrin served as the control. After 30 min at 37 °C and 10 min at 95 °C, the reaction was stopped. According to the protocol of the glucose assay kit, 10 μL of mixture was collected for further testing. The absorbance of the reaction solution at 540 nm was measured, and myrosinase activity was calculated (expressed in U/min·mg).

### 2.4. Measurement of Total Phenolic and Total Flavonoid Contents

Total phenolic content was measured by Folin–Ciocalteu assay [33]. A 1.5 mL of 60% ethanol was used to mix the dry sample powder (0.1 g) and was extracted under 60 °C for 30 min. The mixture was centrifugated under 12,000 rpm at 25 °C for 10 min. Then, 10 μL of supernatant was added into 50 μL of 10% Folin–Ciocalteu’s phenol reagent, 90 μL of distilled water, and 50 μL of 2% sodium carbonate solution. The mixture was stirred and allowed to react under room temperature for 10 min. Furthermore, the absorbance was measured at 760 nm using a microplate reader. The measurements were compared with a calibration curve (mg/g).

A method described by Rao et al. was used to measure the total flavonoid content [34]. Dry sample powder (0.1 g) was mixed with 1.0 mL of 60% ethanol and extracted under 60 °C for 30 min. The mixture was centrifugated under 12,000 rpm at 25 °C for 10 min. Then, 60 μL of supernatant was added into 120 μL of 1 M potassium acetate solution, 30 μL of 10% aluminum nitrite solution, and 90 μL of 60% ethanol. The mixture was allowed to react under 37 °C for 45 min. Then, the absorbance was measured at 470 nm using a microplate reader. The measurements were compared to a rutin calibration curve, and the results were expressed as per gram of dry wight (mg/g DW).

### 2.5. Antioxidant Activities

#### 2.5.1. 2,2-Diphenyl-1-picrylhydrazyl (DPPH) Radical Scavenging Activity

Dry sample powder (50 mg) was mixed with 1.0 mL of ethanol and extracted under 40 °C for 30 min. The mixture was centrifuged under 10,000 rpm, 25 °C for 10 min. Then, 10 μL supernatant was added into the 190 μL of DPPH solution (0.4 mM). The mixture was allowed to react for 10 min in the dark at room temperature. Then, the absorbance was measured at 515 nm. For the blank, ethanol was used instead of the sample. Radical scavenging activity was calculated as follows:

DPPH radical scavenging activity = (1 − (absorbance of the sample/absorbance of the blank)) × 100%

#### 2.5.2. Trolox-Equivalent Antioxidant Capacity (ABTS) Radical Scavenging Activity

A solution of ABTS was dissolved in distilled water in ratios of 1 and 12 mL. The cation (ABTS+) was produced by reacting the diluted ABTS solution with 2.45 mM potassium persulfate and ethanol, and the ratio was 4:76:5 (*v*/*v*/*v*), which was left in the dark for 30 min at room temperature. Dry sample powder (50 mg) was mixed with 1.0 mL of ethanol and was extracted under 40 °C for 30 min. The mixture was centrifuged under 10,000 rpm for 25 °C for 10 min. Then, 10 μL of supernatant was added into 170 μL of ABTS+ solution. The mixture was allowed to react in the dark for 6 min at room temperature. Then, absorbance was measured at 405 nm. For the blank, ethanol was used instead of the sample. Then, radical scavenging activity was calculated as follows:

ABTS radical scavenging activity = (1 − (absorbance of the sample/absorbance of the blank)) × 100%

#### 2.5.3. Ferric-Reducing Antioxidant Power (FRAP)

Ferric-reducing antioxidant power was reflected by the ability to reduce Fe^3+^-2,4,6-tri(2-pyridyl)-1,3,5-triazine (TPTZ) to produce blue Fe^2+^-TPTZ under acidic conduction. The assay was performed according to Benzie and Strain [35]. The Fe^3+^-TPTZ reagent was prepared fresh daily by a 10 mM TPTZ solution in 40 mM HCl, 300 mM acetate buffer (pH 3.6), and 20 mM FeCl_3_·6H_2_O solution with a proportion of 10:1:1 (*v*/*v*/*v*). After preparation, the reagent was warmed at 37 °C for 10 min before use. Fresh sample (0.1 g) was extracted with 1.0 mL of distilled water, and the mixture was centrifugated under 10,000 rpm at 4 °C for 10 min. Then, 6 μL of supernatant was added into the 18 μL of distilled water and 180 μL of Fe^3+^-TPTZ reagent. The mixture was stirred and allowed to react at room temperature for 10 min. The absorbance was measured at 593 nm. For the blank, distilled water was used to replace the sample.

### 2.6. RNA Extraction and Expression Analysis

Total RNA from fresh broccoli florets (0.1 g) was extracted in accordance with a Plant Total RNA Isolation Kit Plus (TIANGEN, Beijing, China). Then, 1 μg total RNA was used to synthesize cDNA following the manufacturer’s instruction of a HiScript III 1st Strand cDNA Synthesis Kit (Vazyme, Nanjing, China). The cDNA samples at 100 ng/μL were used to prepare qPCR reaction solution by using AceQ qPCR SYBR Green Master Mix kit (Vazyme, Nanjing, China), and the qPCR conditions assigned as three stages: stage (i) for predegeneration: 95 °C for 5 min, stage (ii) for 40 circular reactions: 95 °C of degeneration for 10 s and 60 °C of annealing for 30 s, stage (iii) for dissociation curve: the condition is depend on instrument. For the fluorescent qPCR analysis of gene expression, the primers of gene sequences for amplification were designed by National Center for Biotechnology Information (NCBI, Table S1). Actin-2(LOC106315376) was selected for housekeeping gene, the expression of Actin-2 in this representative samples, which were standardized the concentration were used to calibrate, for the differential of Ct value was less than 0.5. Expression data were analyzed by 2^−ΔΔCt^, followed by BioRad Real-time PCR Application Guide.

### 2.7. Statistical Analysis

One-way ANOVA was performed on all data using SPSS 20.0 statistical software (SPSS Inc., Chicago, IL, USA). Duncan tests were performed to observe differences between treatments.

## 3. Results and Discussion

### 3.1. Effects of MeJA on Glucosinolate Biosynthesis in Broccoli Floret, Stem, and Leaves

**Figure 1:**
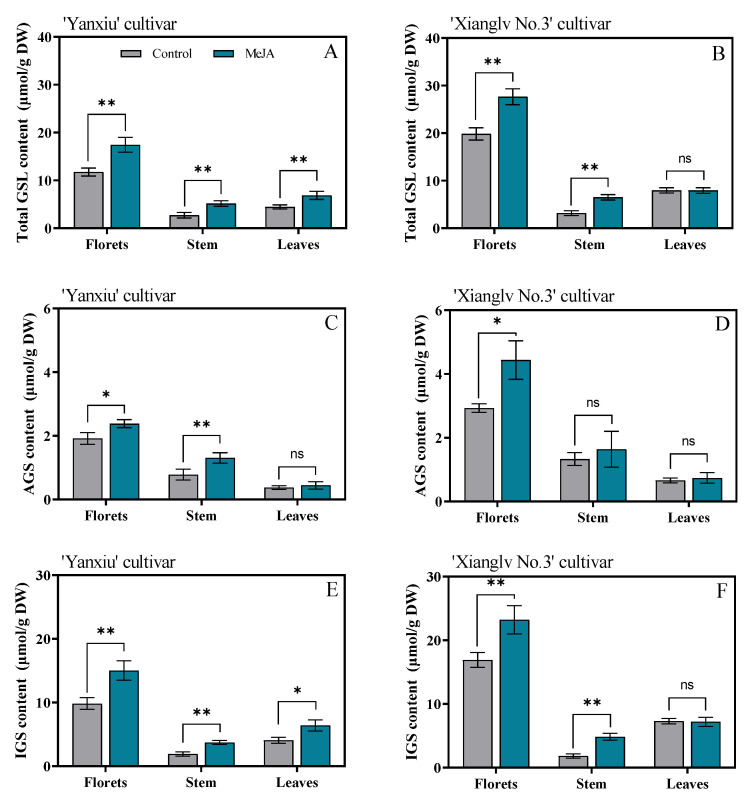
Effects of MeJA on total GSLs (**A**,**B**), AGS (**C**,**D**), and IGS (**E**,**F**) content in broccoli florets, stem, and leaves. GSLs: glucosinolates; AGS: total aliphatic GSLs; IGS: total indole GSLs; DW: dry weight; control: the plants were sprayed with distilled water; MeJA: the plants were sprayed with 40 μmol/L MeJA. The data are displayed with the mean ± SD of three replications. Values with ns, * and ** are significantly different at *p* > 0.05, *p* < 0.05, and *p* < 0.01.

GSLs were characterized with a GSL map and quantified with an internal control (sinigrin). Results are shown in Table S2. Eight kinds of GSLs were isolated by HPLC. Among them, four were AGSs (IBE, SIN, GRA, and ERU), and four were IGSs (4OH, GBC, 4ME, NGBC). The average AGS and IGS content of broccoli florets (2.42 μmol/g DW, 13.37 μmol/g DW) accounted for about 15.3% and 84.7% of the total GSL content (15.80 μmol/g DW) in the control group, respectively (Figure 1 and Table S3). For two cultivars, the content of total GSLs of florets in ‘Xianglv No. 3’ (19.84 μmol/g DW) was higher at about 1.69 folds than that of ‘Yanxiu’ (11.76 μmol/g DW) in the control groups (Figure 1A,B). After MeJA treatment, the total GSL content in broccoli florets, stem, and leaves (except the leaves of ‘Xianglv No. 3’) had considerably increased in two verities compared with the control groups (Figure 1A,B). These results agreed with previous ones, indicating that MeJA treatment remarkably increases GSL content in other Brassicaceae vegetables, such as pak choi [1], cauliflower [18], cabbage [36], and leaf mustard [37]. Moreover, the total GSLs content in ‘Yanxiu’ florets (17.43 μmol/g DW) increased remarkably by 48.2% after MeJA treatment than the control groups (11.76 μmol/g DW), whereas the value increased substantially by 39.4% in ‘Xianglv No. 3’ (27.66 μmol/g DW vs. 19.84 μmol/g DW). A higher promoted effect was observed in the low-GSL cultivar (Yanxiu) than in the high-GSL ones (Xianglv No. 3), which indicates that total GSL content depends remarkably on the cultivar [38].

A similar phenomenon was observed in the two cultivars. The GSL contents (including AGS and IGS, 15.80 μmol/g DW) of florets were higher than those of stems (2.94 μmol/g DW) at about 5.37 folds and leaves (6.20 μmol/g DW) at 2.55 folds, which indicates that a floret was the primary organ involved in GSL biosynthesis or storage (Figure 1A,B). MeJA treatment remarkably increased the AGS and IGS contents of florets (Figure 1C–F). A previous study [39] agreed with our result, which showed that MeJA treatment induced a remarkable increase in the IGS content of four pak choi cultivars, but AGS increased in only one cultivar, indicating that MeJA treatment had less effect on AGS content than IGS. Brown et al. [40] also reported that AGS biosynthesis depends on genetics rather than environmental factors, in which AGS biosynthesis is perhaps due to limited response to biotic stress. However, AGS content in broccoli floret can also remarkably change with MeJA treatment in the present study [1,41]. The result showed that the AGS and IGS contents (3.41 μmol/g DW, 19.13 μmol/g DW) of broccoli florets increased by 40.7% and 43.1%, respectively, after MeJA treatments compared with the control groups (2.42 μmol/g DW, 13.37 μmol/g DW) (Figure 1C–F).

### 3.2. Effects of MeJA on Glucoraphanin, Sulforaphane Content, and Myrosinase Activity in Broccoli Florets, Stem, and Leaves

**Figure 2:**
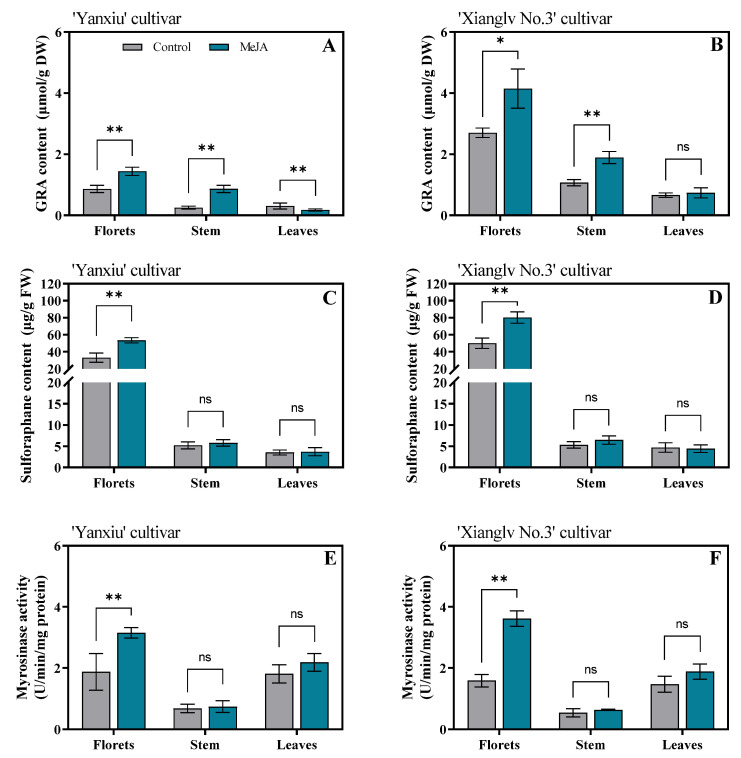
Effects of MeJA on GRA content(**A**,**B**), sulforaphane content (**C**,**D**), and myrosinase activity (**E**,**F**) in broccoli florets, stem, and leaves. GRA: Glucoraphanin, DW: dry weight, FW, fresh weight. Control: the plants were sprayed with distilled water, MeJA: the plants were sprayed with 40μmol/L MeJA. The data are displayed with the mean ± SD of three replications. Values with ns, * and ** are significantly different at *p* > 0.05, *p* < 0.05 and *p* < 0.01.

The GRA contents, sulforaphane contents, and myrosinase activity of broccoli florets, leaves, and stem are shown in Figure 2. GRA is commonly converted into sulforaphane owing to the action of myrosinase upon tissue disruption [42]. The accumulation of GRA, as a sulforaphane precursor, can be promoted through agricultural practices can effectively increase the sulforaphane content in broccoli. The GRA contents of florets in the two cultivars remarkably increased after MeJA treatment. These results agreed with previous studies on other Brassicaceae vegetables [1,43]. However, no remarkable difference was observed in some reports [20,44,45]. The possible reason was that the GRA-regulating role of exogenous MeJA depends on genotype, edible parts, and treatment concentration. A similar remarkable increase was observed for GRA content in broccoli stem in the two cultivars after MeJA treatment. However, the GRA content in broccoli leaves in ‘Yanxiu’ markedly decreased after MeJA treatment, whereas no substantial change in ‘Xianglv No. 3’ (Figure 2A,B). After MeJA treatment, myrosinase activity had a similar increasing trend as the sulforaphane content of florets in two cultivars (Figure 2C–F). Correlation analysis showed that sulforaphane had significantly positive correlations with GRA contents and myrosinase activity (r = 0.896, *p* < 0.01; r = 0.754, *p* < 0.01) (Table S5). As a result, sulforaphane content increased due to the GRA biosynthesis promoting and myrosinase activity inducing by exogenous MeJA treatment. Similar results were reported by Ku et al. [19]. Myrosinase enzymes play a key roles in the hydrolysis of GRA into bioactive and anticarcinogen products, such as sulforaphane [46]. Although exogenous MeJA treatment remarkably affects the GRA content of broccoli stem and leaves in two cultivars, it had no substantial effect on sulforaphane content (Figure 2A–D). No considerable effect was observed in the myrosinase activities of broccoli stem and leaves in the two cultivars (Figure 2E,F). Therefore, the reason for the unremarkable effect of exogenous MeJA treatment on the sulforaphane contents of broccoli stem and leaves was the lower responding level of myrosinase activity under MeJA treatment. Thus, sulforaphane accumulation depends on high GRA biosynthesis efficiency and myrosinase activity [47].

### 3.3. Effects of MeJA on Gene Expression in Relation to the Glucosinolate Metabolism of Broccoli Florets

**Figure 3:**
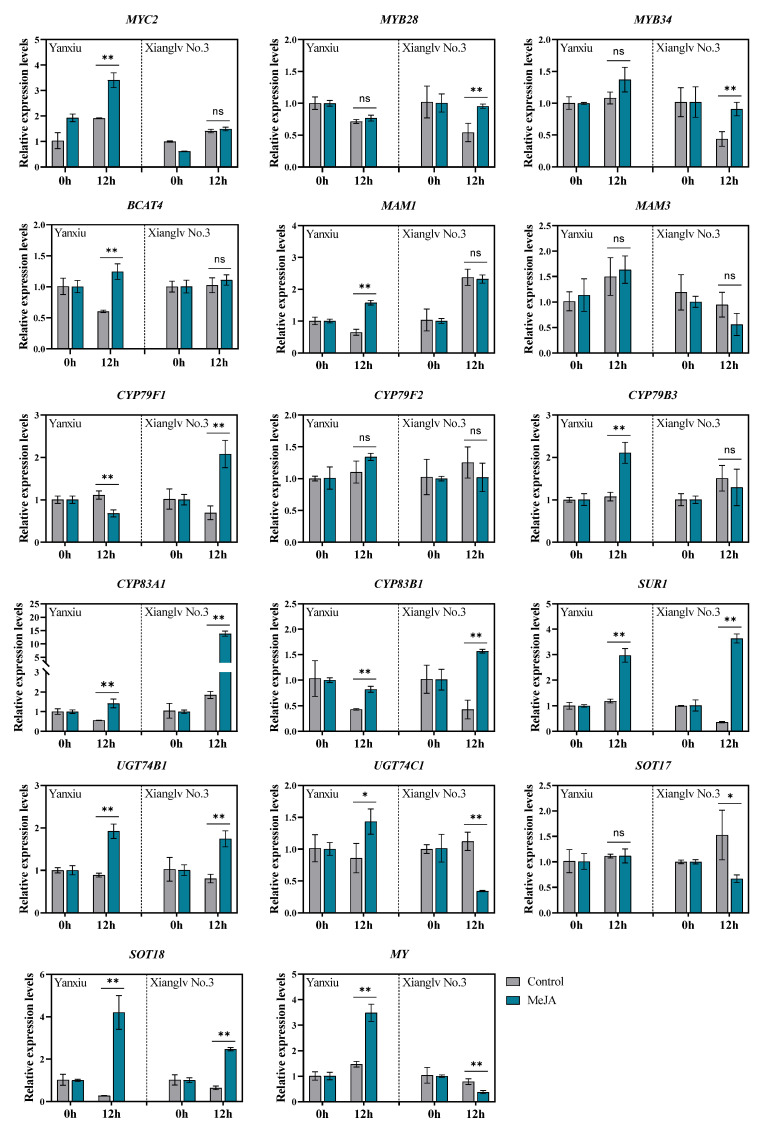
Relative expression of genes involved in GSLs metabolism under MeJA treatments. The data were analyzed using the 2^−ΔΔCt^ method with control groups as 1; Control: the plants were sprayed with distilled water; MeJA: the plants were sprayed with 40 μmol/L MeJA. The data are displayed with the mean ± SD of three replications. Values with ns, * and ** are significantly different at *p* > 0.05, *p* < 0.05, and *p* < 0.01. For the full names of genes see Table S1.

The relative expression levels of 17 genes involved in GSL metabolism were determined by RT-qPCR to understand the gene expression pattern of the two broccoli cultivars floret in response to exogenous MeJA treatment after 12 h. The results of gene-expression analysis are shown in Figure 3. Notably, 10 genes were significantly upregulated by MeJA treatment based on log_2_Fold Change > 1 and *p* < 0.05 and the differential expression pattern was observed between these two cultivars.

For AGS biosynthesis, the genes expression of *CYP83A1*, *SUR1*, *UGT74B1* and *SOT18* were remarkably induced in both two cultivars after 12 h MeJA treatment when compared with the control groups, particularly, the expression of *BCAT4*, *MAM1*, and *UGT74C1* in *‘*Yanxiu’ cultivar and CYP79F1 in *‘*Xianglv No.3’ cultivar were upregulated significantly. The expression of *CYP83A1* was upregulated for 2.53 folds (Yanxiu) and 7.47 folds (Xianglv No. 3), meanwhile, *SUR1* for 2.50 folds (Yanxiu) and 10.1 folds (Xianglv No. 3) and *SOT18* for 15.4 folds (Yanxiu) and 3.83 folds (Xianglv No. 3). For IGS biosynthesis, the expression of *CYP83B1* (1.91 folds of ‘Yanxiu’, 3.65 folds of ‘Xianglv No. 3’) and *UGT74B1* (2.17 folds of ‘Yanxiu’, 2.16 folds of ‘Xianglv No. 3’) were higher after MeJA treatment. Transcription factors are the crucial point for exogenous MeJA treatment response to transcribe the downstream target gene expression. R2R3-MYB transcription factors MYB28 and MYB34 play important role in aliphatic and indolic-GSL biosynthesis, respectively. Furthermore, the bHLH transcription factor MYC2 is a major positive regulator of JA pathway, which can response to MeJA treatment regulates glucosinolate synthesis [48]. Interestingly, the expression levels of *MYB28* (1.07 folds) and *MYB34* (1.27 folds) just remarkable induced in ‘Xianglv No. 3’, and *MYC2* (1.78 folds) was induced in ‘Yanxiu’ (log2Fold Change < 1) after MeJA treatment when compared with control groups. For the degradation pathway of GSL, the *MY* gene, obtained differential expression pattern between both cultivars, which was significant induced in ‘Yanxiu’ and suppressed in ‘Xianglv No. 3’.

These results indicated that just 40 µM concentration of MeJA treatment can effectively induce AGS and IGS biosyntheses. Similarly, a 250 µM MeJA treatment can remarkably increase *MYB28*, *MYB34*, *MAM3*, *SUR1*, *SOT17*, *SOT18*, *TGG1*, and *TGG2* expression in kale [45], whereas a 400 µM MeJA treatment to broccoli florets can substantially increase the expression of *SOT17*, *SOT18*, *TGG1*, and *TGG2* [49]. The results showed the effect of exogenous MeJA treatment on GSL biosynthesis by inducing gene expression, but it varies depending on plant genotype and treatment concentration. MYC2 is a major positive regulator of JA pathway and can promote JA-induced IGS biosynthesis by positively regulating the JA-dependent regulator, MYB34 [48]. In the present study, IGS content was remarkably increased in broccoli florets, and *MYC2* was upregulated under MeJA treatment.

### 3.4. Effects of MeJA Treatment on Total Phenolics and Total Flavonoids Content of Broccoli Florets, Stem, and Leaves

**Figure 4:**
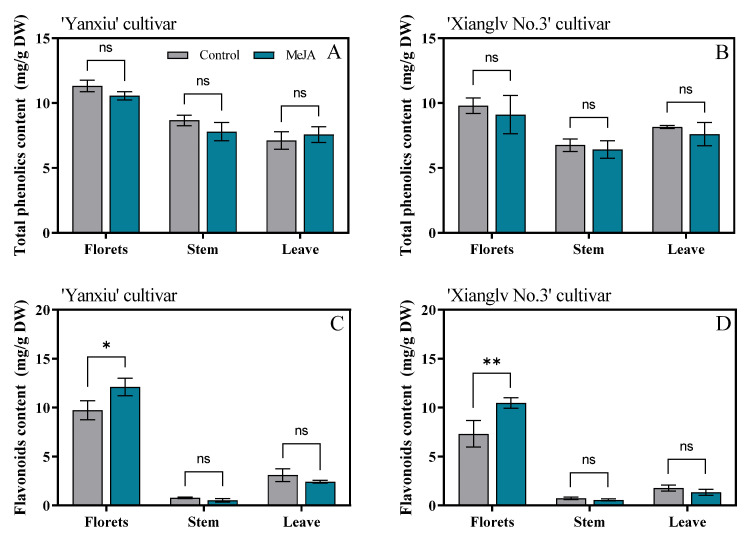
Effects of MeJA on total phenolics (**A**,**B**) and flavonoids content (**C**,**D**) in broccoli florets, stem, and leaves. DW: dry weight; Control: the plants were sprayed with distilled water; MeJA: the plants were sprayed with 40 μmol/L MeJA. The data are displayed with the mean ± SD of three replications. Values with ns, * and ** are significantly different at *p* > 0.05, *p* < 0.05 and *p* < 0.01.

The total phenolic contents of broccoli florets in the two cultivars were higher than those in stem and leaves. However, the total phenolic content of exogenous MeJA-treated broccoli had no remarkable effect than the control in florets, stem, and leaves (Figure 4A,B). Guan et al. [50] reported that total phenolic content had no substantial change within 12 h after 1 μ or 10 μM MeJA treatment on broccoli, but had a considerable increase after 100 μM MeJA treatment. A similar result was found by Villarreal-García et al. [51]. They indicated that the effect of MeJA on total phenolics depends on concentration; the mechanism of resistance to exogenous stress could not been induced by a low MeJA concentration [49].

In our study, 40 µM MeJA treatment remarkably increased the flavonoid contents of broccoli florets in two cultivars; however, no substantial effect was observed in broccoli stems and leaves (Figure 4C,D). Similar to our results, 0.5 mM MeJA treatment increased the flavonoid content of hydroponically grown pak choi [1], and a low MeJA dose (10 µM) increased the flavonoid content of broccoli sprouts by about 31% for 7 days [52].

### 3.5. Effects of MeJA Treatment on Antioxidant Activities of Broccoli Florets, Stem, and Leaves

**Figure 5:**
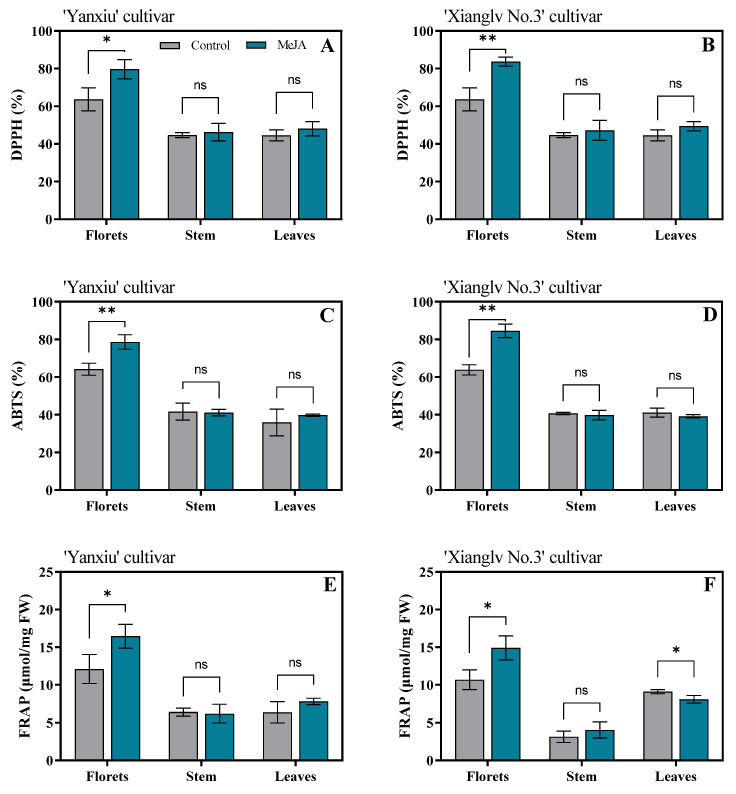
Effects of MeJA on DPPH(**A**,**B**), ABTS (**C**,**D**), and FRAP (**E**,**F**) in broccoli florets, stem, and leaves. DPPH: 2,2-di-phenyl-1-picrylhydrazyl radical scavenging capacity; ABTS: trolox-equivalent antioxidant capacity; FRAP: ferric-reducing antioxidant power; FW: fresh weight; Control: the plants were sprayed with distilled water; MeJA: the plants were sprayed with 40 μmol/L MeJA. The data are displayed with the mean ± SD of three replications. Values with ns, * and ** are significantly different at *p* > 0.05, *p* < 0.05, and *p* < 0.01.

The results showed that exogenous MeJA treatment remarkably increased the antioxidant activities of broccoli florets in the two cultivars. The antioxidant activities showed an increasing trend in three methods (DPPH radical scavenging capacity, ABTS, and FRAP) and had the same trends with flavonoids (Figure 5). The highest scavenging capacity was found in broccoli florets treated with MeJA. However, MeJA treatment caused no remarkable changes in broccoli stems and leaves.

Polyphenols are major plant compounds with antioxidant activity [53]. Sokół-Łetowska et al. [54] also noted that polyphenols have an ideal chemical structure for scavenging free radicals. However, exogenous MeJA treatment had no remarkable effect on the total phenolics of the two broccoli cultivars (Figure 4A,B). In addition to polyphenols, the higher antioxidant capacity of broccoli treated with MeJA may be due to the considerably higher contents of GSL, sulforaphane, and flavonoids (Figure 2 and Figure 4). The bioactivity of GSLs and their hydrolysis products in cruciferous vegetables have been reported [55]. Therefore, compared with the control group, exogenous MeJA treatment may be beneficial in improving antioxidant activity.

### 3.6. The Overall Improvement of MeJA Treatment on Broccoli Florets Based on Principal Component Analysis and Pearson Correlation Analysis

**Figure 6:**
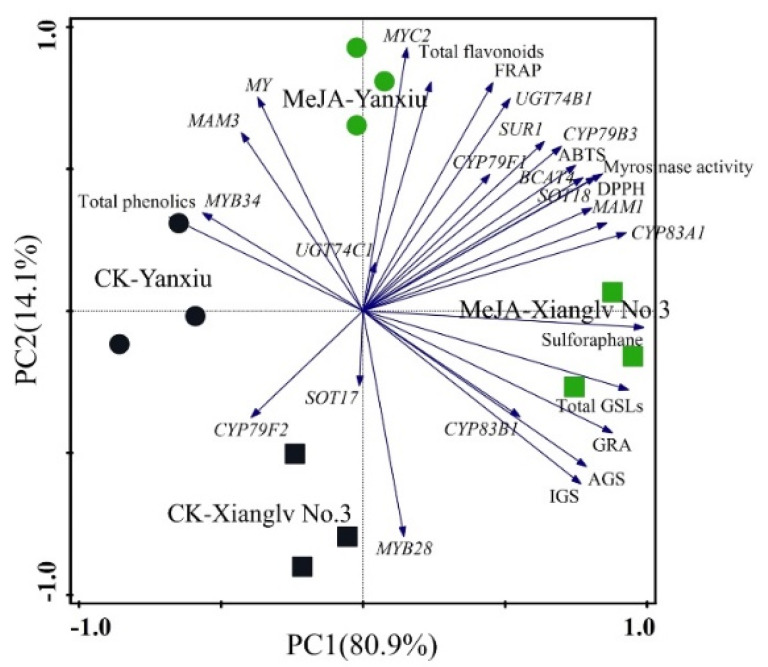
Biplot of the principal component analysis of the observed parameters of broccoli florets of two cultivars with or without the treatment of MeJA. “•”: broccoli florets of ‘Yanxiu’ without MeJA treatment; “•”: broccoli florets of ‘Yanxiu’ with MeJA treatment; “■”: broccoli florets of ‘Xianglv No.3’ without MeJA treatment; “■”: broccoli florets of ‘Xianglv No.3’ with MeJA treatment. GSLs: glucosinolates, AGS: total aliphatic GSLs; IGS: total indole GSLs, GRA: Glucoraphanin, DPPH: 2,2-di-phenyl-1-picrylhydrazyl radical scavenging capacity; ABTS: trolox-equivalent antioxidant capacity; FRAP: ferric-reducing antioxidant power.

**Figure 7:**
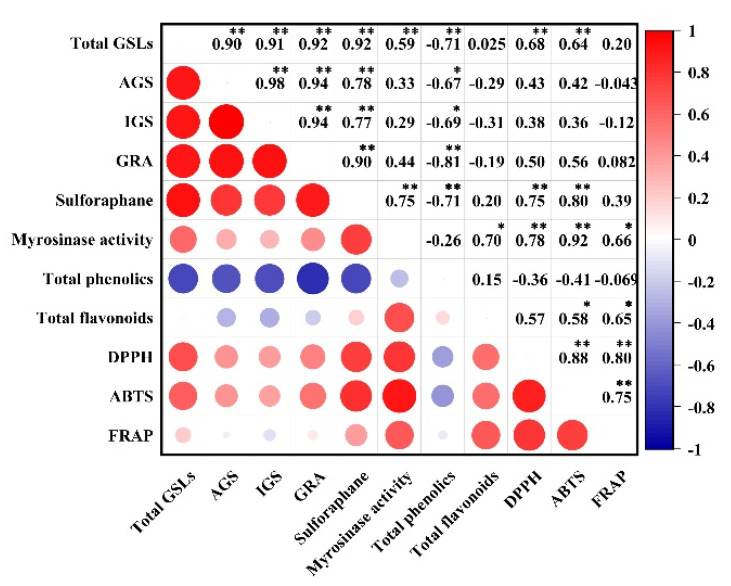
Pearson correlation analysis of bioactive substances and antioxidant activity broccoli florets of two cultivars. GSLs: glucosinolates; AGS: total aliphatic GSLs; IGS: total indole GSLs; GRA: glucoraphanin; DPPH: 2,2-di-phenyl-1-picrylhydrazyl radical scavenging capacity; ABTS: trolox-equivalent antioxidant capacity; FRAP: ferric-reducing antioxidant power. * and ** represent significant correlation at the 0.05 and 0.01 level (2-tailed), respectively.

Broccoli florets are the main edible part, and higher levels of bioactive substances were detected in the broccoli florets of two cultivars than in other parts. Therefore, studying the effects of the observed parameters on the comprehensive quality of broccoli and their correlation by principal component analysis is necessary. Thirty indexes of bioactive substances, antioxidant activities, and gene expression involved in GSL biosynthesis were analyzed in the florets of two broccoli cultivars, and the results were shown in Figure 6. According to the above parameters, principal component 1 (PC1), and principal component 2 (PC2) accounted for about 95.0% of the total variance. PC1 showed about 80.9% of the total variance and was positively correlated to GSLs, sulforaphane, myrosinase activity, flavonoids, antioxidant activities (DPPH, ABTS, and FRAP), and some gene expression (*MYC2*, *MYB28*, *BCAT4*, *MAM1*, *CYP79F1*, *CYP83A1*, *CYP83B1*, *CYP83B3*, *SUR1*, *UGT74B1*, *UGT74C1*, and *SOT18*). PC2 accounted for about 14.1% of the total variance; represented variances; and was positively correlated with myrosinase activity, total phenolics, total flavonoids, antioxidant activities, and gene expression (*MYC2*, *MYB34*, *BCAT4*, *MAM1*, *MAM3*, *CYP79F1*, *CYP83A1*, *CYP83B3*, *SUR1*, *UGT74B1*, *UGT74C1*, *SOT18*, and *MY*). These parameters were higher in broccoli floret with MeJA treatment than those in groups. Overall, preharvest MeJA treatment showed a positive effect on the measured indexes for two broccoli cultivars. Hence, preharvest supplementation with 40 μM MeJA could be a good way to improve the antioxidant properties of broccoli florets. Moreover, Pearson correlation analysis showed that the significant positive correlation between bioactive substances and antioxidant activity, especially sulforaphane and flavonoids (Figure 7).

## 4. Conclusions

In this study, we investigated the effects of preharvest treatment with exogenous MeJA (40 μΜ) on GSL biosynthesis, sulforaphane accumulation, and antioxidant activity in broccoli florets, stem, and leaves. The overall results revealed that GSL biosynthesis and sulforaphane accumulation were most likely induced by exogenous MeJA treatment by upregulating the expression of *CYP83A1*, *SUR1*, *UGT74B1*, and *SOT18* genes. Exogenous MeJA treatment more remarkably contributed to the increase in GSL biosynthesis in broccoli cultivars with low-level GSL content (Yanxiu) than that with high-level GSLs (Xianglv No.3). Moreover, MeJA treatment had a more substantial increasing effect in broccoli florets than in stem and leaves. Interestingly, total flavonoid content substantially increased in broccoli florets after MeJA treatment, but total phenolics did not. These results strongly support the finding that preharvest MeJA treatment remarkably enhanced the antioxidant activities (DPPH, ABTS, FRAP) of broccoli florets. In conclusion, MeJA mediated bioactive compound metabolism, had positive effects on GSL biosynthesis, sulforaphane, and flavonoids accumulation. There was a significant positive correlation between bioactive substances and antioxidant activity, especially sulforaphane and flavonoids. Hence, preharvest supplementation with 40 μM MeJA could be a good option to improve the antioxidant properties of broccoli floret.

## Data Availability

Data is contained within the article or supplementary material.

## Supplementary Materials

The following supporting information can be downloaded at: https://www.mdpi.com/article/10.3390/antiox11071298/s1, Table S1: List of primers used for RT-qPCR in broccoli; Table S2: PrimDesulphated GSLs identified in broccoli seedlings leaves using UPLC–MS.
