# Supplementary material for: Preharvest Methyl Jasmonate Treatment Increased Glucosinolate Biosynthesis, Sulforaphane Accumulation, and Antioxidant Activity of Broccoli
Source: Antioxidants (Basel). 2022 Jun 29;11(7):1298. doi: 10.3390/antiox11071298 (PMC9312100; doi:10.3390/antiox11071298)
Supplement: Supplementary file 1 [file antioxidants-11-01298-s001.zip › antioxidants-1762406-supplementary.pdf]

Table S1 List of primers used for RT-qPCR in broccoli

Function	Gene Name	Protein name		Sequence (5’ to 3’)	Gene ID *
Housekeeping gene	Actin-2		F	GGAGCTGAGAGATTCC	LOC106315376
			R	GAACCACCACTGAGGA[illegible]	
Transcription factor	MYC2	Transcription factor MYC2	F	GCAAGGAGGAGTGTTT	LOC106295384
			R	ATCGTTCTCGCCTTGAT	
	MYB28	Transcription factor MYB28	F	CCACCACGCAGCCAGA	LOC106301630
			R	TCAGGGAAGCCATTGG	
	MYB34	Transcription factor MYB34	F	CTTCATGCTCTTATGGG	LOC106322588
			R	CTTCTTGAGATTTGTGT	
Side-chain elongation	*BCAT4*	Branched-chain amino acid aminotransferase 4	F	ATGTTTTGTTTTTGGAT	LOC106292635
			R	CGATGACACTTTTACGT	
	MAM1	Methylthioalkylmalate synthase 1	F	CTTCACTGACTCCCAA	LOC106324637
			R	AAACCATCAAAACCAT	
	MAM3	Methylthioalkylmalate synthase 3	F	TAGGTGAGAGAAGTGG	LOC106321871
			R	CTATGGGTTTATGTGGT	
Core structure synthesis	*CYP79F1*	Cytochrome P450 79F1	F	AACCTCCTTGCTTACCT	LOC106343089
			R	TTGTCCTAACCTTCCCT	
	CYP79F2	Cytochrome P450 79F2	F	CTGGTCGAAAGTGAAT	LOC103872429
			R	TTTGGATAAAGGTTGG	
	CYP79B3	Tryptophan N- monooxygenase 2	F	CCCTTTGCTTACCGCTG	LOC103864341
			R	CTTTCTTTGCCGACGAC	
	CYP83A1	Cytochrome p450, family 83, subfamily a, polypeptide 1	F	TTCCCGTTATTCGGATT	LOC106342466
			R	CTCTCAGTTTCTGGCTT	
	CYP83B1	CYP83B1 monooxygenase	F	ACACTTCCTCTTTCGTC	LOC103833295
			R	CGGTTCCGTACTCGTTA	
	SUR1	S-alkyl-thiohydroximate lyase SUR1	F	GACATAACACCTGACC	LOC106306752
			R	CCTTGAGCCTATCACAC	
	UGT74B1	UDP- glycosyltransferase 74B1	F	CGAATCAAAGACGATA	LOC103840711
			R	ACCAAACGAAACAAAA	
	UGT74C1	UDP- glycosyltransferase 74C1	F	ATGATCTGCCTTCTTTC	LOC106342937
			R	ACTTTGCTGGTTTATTC	
	SOT17	Cytosolic sulfotransferase 17	F	AAGGCTGGAGACCGAA	LOC106343839
			R	TTCAGGAGAGGGTTGG	
	SOT18	Cytosolic sulfotransferase 18	F	CGAAGGAGGAAGAGG	LOC106336556
			R	TGGCATAAACAGCAGG	
GSL degradation	*MY*	Myrosinase 1	F	ACCAAATACGGCGACC	LOC106326800
			R	TCCAAGAGCCCATGCA	

Note: * identified from the Brassica oleracea var. oleracea cultivar TO1000 genome data base.
The genes were reported in reference “A Comprehensive Gene Inventory for Glucosinolate Biosynthetic Pathway in *Arabidopsis thaliana*”.

Table S2 Desulphated GSLs identified in broccoli florets, stem, and leaves using UPLC–MS.

No.	Retention time (min)	Trivial name	Abbreviations	Semisystematic name	Molecular weight[a]
1	8.3	Glucoiberin	IBE	3-Methylthioallylthio GLS	343
2	10.6	Sinigrin	SIN	2-Propenyl	279
3	11.2	Glucoraphanin	GRA	4-Methylsulfinylbutyl GLS	357
4	18.5	4-hydroxyglucobrassicin	4OH	4-Hydroxyindol-3-ylmethyl GLS	384
5	24.72	Glucoerucin	ERU	4-Methylsulthiobutyl GLS	351
6	27.09	Glucobrassicin	GBC	Indole-3-methylthio GLS	368
7	30.16	4-Methoxyglucobrassicin	4ME	4-Methoxyinol-3- ylmethyl GLS	398
8	33.62	Neoglucobrassincin	NGBC	1-Methoxyindol-3-ylmethyl GLS	398

[a] As desulfo- glucosinolate

Table S3 Effects of MeJA on GSLs content in broccoli florets, stem and leaves.

Cultivars	Organ/ Tissue	Control			MeJA		
		Total GSLs content (μmol/g DW)	Aliphatic GSLs content (μmol/g DW)	Indole GSLs content (μmol/g DW)	Total GSLs content (μmol/g DW)	Aliphatic GSLs content (μmol/g DW)	Indole GSLs content (μmol/g DW)
Yanxiu	Florets	11.76±0.84	1.92±0.18	9.84±0.93	17.43±1.55	2.38±0.13	15.04±1.53
	Stem	2.70±0.59	0.78±0.17	1.92±0.34	5.17±0.57	1.31±0.16	3.73±0.31
	Leaves	4.44±0.43	0.38±0.062	4.07±0.47	6.87±0.86	0.45±0.12	6.42±0.88
Xianglv NO.3	Florets	19.84±1.29	2.93±0.13	16.91±1.18	27.66±1.66	4.44±0.61	23.22±2.22
	Stem	3.17±0.53	1.33±0.20	1.84±0.33	6.51±0.59	1.64±0.56	4.87±0.55
	Leaves	7.96±0.56	0.66±0.073	7.30±0.44	7.95±0.58	0.74±0.16	7.21±0.71

Table S4 Effects of MeJA on glucoraphanin content, sulforaphane content and myrosinase activity in broccoli florets, stem and leaves.

Cultivars	Organ/ Tissue	Control			MeJA		
		Glucoraphanin content (μmol/g DW)	Sulforaphane content (μg/g DW)	Myrosinase activity (U/min/mg protein)	Glucoraphanin content (μmol/g DW)	Sulforaphane content (μg/g DW)	Myrosinase activity (U/min/mg protein)
Yanxiu	Florets	0.86±0.12	33.05±5.48	1.87±0.60	1.44±0.14	53.36±3.05	3.15±0.17
	Stem	0.25±0.05	5.21±0.83	0.68±0.14	0.87±0.12	5.78±0.76	0.74±0.19
	Leaves	0.31±0.096	3.53±0.61	1.81±0.30	0.18±0.034	3.70±0.95	2.19±0.29
Xianglv NO.3	Florets	2.70±0.16	49.97±6.15	1.59±0.20	4.15±0.64	80.28±6.70	3.61±0.25
	Stem	1.07±0.10	5.31±0.77	0.54±0.14	1.89±0.20	6.45±0.97	0.64±0.019
	Leaves	0.66±0.073	4.71±1.11	1.47±0.26	0.74±0.16	4.42±0.90	1.88±0.25

Table S5 Correlation analysis of sulforaphane content between glucoraphanin contents and myrosinase activity in broccoli florets

		Sulforaphane content	glucoraphanin contents	myrosinase activity
Sulforaphane content	Pearson correlation	1.000	0.896**	0.754**
	Significant (2-tailed)		0.000	0.005
	Sample number	12	12	12
Glucoraphanin contents	Pearson correlation	0.896**	1.000	0.444
	Significant (2-tailed)	0.000		0.148
	Sample number	12	12	12
Myrosinase activity	Pearson correlation	0.754**	0.444	1.000
	Significant (2-tailed)	0.005	0.148	
	Sample number	12	12	12

**. Correlation is significant at the 0.01 level (2-tailed)

Table S6 Effects of MeJA on glucoraphanin content, sulforaphane content and myrosinase activity in broccoli florets, stem and leaves.

Cultivars	Organ/ Tissue	Control		MeJA	
		Total phenolics content (mg/g DW)	Total flavonoids Content (mg/g DW)	Total phenolics content (mg/g DW)	Total flavonoids Content (mg/g DW)
Yanxiu	Florets	11.32±0.44	9.73±0.97	10.56±0.32	12.10±0.89
	Stem	8.66±0.41	0.77±0.067	7.80±0.71	0.52±0.18
	Leaves	7.11±0.68	3.09±0.65	7.57±0.60	2.42±0.14
Xianglv No.3	Florets	9.79±0.60	7.31±1.36	9.11±1.48	10.47±0.53
	Stem	6.75±0.48	0.73±0.14	6.42±0.68	0.56±0.11
	Leaves	8.15±0.12	1.77±0.32	7.60±0.89	1.33±0.31

Table S7 Effects of MeJA on glucoraphanin content, sulforaphane content and myrosinase activity in broccoli florets, stem and leaves.

Cultivars	Organ/ Tissue	Control			MeJA		
		DPPH (%)	ABTS (%)	FRAP (μmol/mg FW)	DPPH (%)	ABTS (%)	FRAP (μmol/mg FW)
Yanxiu	Florets	63.63±6.11	64.17±2.49	12.10±1.94	79.62±5.06	78.59±3.83	16.46±1.57
	Stem	44.67±1.40	41.72±4.50	6.40±0.52	46.24±4.61	41.17±1.71	6.20±1.23
	Leaves	44.49±2.86	35.93±7.13	6.37±1.40	48.04±3.77	39.77±0.57	7.80±0.41
Xianglv No.3	Florets	66.40±2.52	63.86±2.69	10.67±1.31	83.67±2.38	84.57±3.59	14.92±1.58
	Stem	48.46±3.07	40.71±0.56	3.14±0.74	47.17±5.27	39.84±2.50	4.03±1.07
	Leaves	52.81±0.82	41.17±2.38	9.09±0.27	49.37±2.49	39.21±0.85	8.08±0.50
